# Influence of Botanical Origin and Chemical Composition on the Protective Effect against Oxidative Damage and the Capacity to Reduce In Vitro Bacterial Biofilms of Monofloral Honeys from the Andean Region of Ecuador

**DOI:** 10.3390/ijms19010045

**Published:** 2017-12-23

**Authors:** Marilyn García-Tenesaca, Eillen S. Navarrete, Gabriel A. Iturralde, Irina M. Villacrés Granda, Eduardo Tejera, Pablo Beltrán-Ayala, Francesca Giampieri, Maurizio Battino, José M. Alvarez-Suarez

**Affiliations:** 1 Facultad de Ingeniería y Ciencias Agropecuarias, Universidad de Las Américas, Quito 170125, Ecuador; mmgarcia@udlanet.ec (M.G.-T.); enavarrete@udlanet.ec (E.S.N.); 2 Laboratorios de Investigación, Universidad de Las Américas, Quito 170125, Ecuador; gabriel.iturralde@udla.edu.ec (G.A.I.); irina.villacres@udla.edu.ec (I.M.V.G.); 3 Facultad de Ingeniería y Ciencias Agropecuarias, Grupo de Bioquimioinformática, Universidad de Las Américas, Quito 170125, Ecuador; eduardo.tejera@udla.edu.ec; 4 Colegio de Administración y Economía, Universidad San Francisco de Quito, Cumbayá, Quito 170157, Ecuador; pbeltran@usfq.edu.ec; 5 Dipartimento di Scienze Cliniche Specialistiche ed Odontostomatologiche (DISCO)-Sez. Biochimica, Facolta di Medicina, Università Politecnica delle Marche, 60121 Ancona, Italy; f.giampieri@univpm.it; 6 Escuela de Medicina Veterinaria y Zootecnia, Grupo de Investigación en Biotecnología Aplicada a Biomedicina (BIOMED), Universidad de Las Américas, Quito 170125, Ecuador

**Keywords:** Ecuadorian monofloral honey, avocado honey, Eucalyptus honey, Rapeseed honey

## Abstract

Three types of monofloral honey from the Andean regions of Ecuador (Avocado, Eucalyptus, and Rapeseed honey) were analyzed to determine their floral origin, physicochemical parameters, chemical composition, antioxidant capacity, and their capacity to reduce in vitro bacterial biofilms. The chemical composition varied considerably depending on floral origin. The highest values of bioactive compounds were found in Avocado honey, classified as dark amber in color, while the lowest values were found in Eucalyptus honey followed by Rapeseed honey, both classified as extra light amber. When compared to Eucalyptus and Rapeseed honey, Avocado honey showed a more effective superoxide scavenging activity, chelating metal ions capacity, and a higher ability to protect human erythrocyte membranes against lipid peroxidation. For antimicrobial activity, the hydrogen peroxide content and the capacity to inhibit the biofilm formation, and to remove preformed biofilm from *Staphylococcus aureus* and *Klebsiella pneumoniae* was determined. Avocado honey showed the highest values of hydrogen peroxide content, as well as the highest capacity to reduce in vitro bacterial biofilms. A correlation between color vs. phenolics content vs. superoxide scavenging activity vs. chelating metal ions capacity, and the capacity to protect human erythrocyte membranes against lipid peroxidation was found.

## 1. Introduction

Humankind has used honey since ancient times, firstly attracted by its sweet taste, and subsequently by the medical properties that have been attributed to it throughout the ages. Honey is a supersaturated solution of sugar that is produced by *Apis mellifera* bees from the nectar collected from flowers, secretions of living parts of plants, or excretions of plant-sucking insects. These secretions are collected by bees and transformed by combining them with specific substances from their own deposits and leaving them in honeycombs to ripen and mature (EU Council, 2002). From a chemical point of view, honey is considered a supersaturated solution of sugars, with fructose (38%) and glucose (31%) being the two most abundant components. Other minor compounds have also been associated with honey’s biological properties, such as polyphenols, enzymes, free amino acids, proteins, minerals, and vitamins [1].

The chemical composition of honey makes it a complex natural mixture of chemical compounds allowing it to show important biological properties, such as the ability to promote wound healing [2] and its antimicrobial, anti-inflammatory, and antioxidant capacity [3]. The antioxidant and antimicrobial capacity of honey has been one of the biological properties most studied in honey and has been closely associated with its floral origin [4]. The antioxidant capacity has been associated with its content of antioxidant compounds, such as polyphenols [3], while its antimicrobial properties have been mainly associated with its osmotic properties, as well as the presence of hydrogen peroxide and of other minor non-peroxide compounds, such as polyphenols and a particular protein, known as defensin-1 [2,5].

The composition of honey depends on several factors- mainly its floral origin and geographical conditions [6], which are reflected in its quality and biological properties, hence the importance of studying the characteristics of honey produced in different regions of origin. A unique and interesting case is Ecuador. Being considered as one of the most biodiverse countries in the world, Ecuador possesses a floral wealth that can be widely used for honey production, however, to date, there is little information about the quality, chemical composition, and biological properties of the honey that is produced in this country. So, the aim of this study was to confirm the floral origin, as well as determine the physicochemical parameters, antioxidant capacity, and antimicrobial activity of three types of monofloral honey that is produced in the Andean region of Ecuador. Moreover, a possible relationship between floral origin, chemical composition, and biological properties was also determined.

## 2. Results and Discussion

### 2.1. Physicochemical Analyses

Table 1 shows the results of the physicochemical analysis determined in the three monofloral honey types studied. According to the colour analysis, Avocado honey could be classified as dark amber (values > 114 mm Pfund), which is in line with previously reported values for this honey type [7,8], while Eucalyptus honey could be classified as extra light amber (values > 34 < 50 mm Pfund). Colour values in the Ecuadorian Eucalyptus honey were lower than those previously reported in other regions, such as Eucalyptus honey from the northwest Iberian Peninsula, classified as light amber [9], or Italian Eucalyptus honey, classified as light amber [10]. Rapeseed honey was classified as extra light amber, which is within the range of the colour values previously reported for this monofloral honey [10,11,12].

Moisture content in honey from *Apis mellifera* is well defined in international quality standards [13]. Since high moisture content can affect honey quality and its organoleptic properties and biological activity [14], the moisture content in honey samples was analyzed and compared with international standards [13]. According to the results here exposed, all of the honey samples were within the permissible range for honey from tropical regions (≤20). Moisture content in honey is related to environmental and geographical conditions, apiary management, and honey storage, so the results here exposed suggest appropriate handling and storage conditions of honey by beekeepers.

Honey is known for its acidic nature, however at present the pH ranges for *Apis mellifera* honey have not been established, hence the importance of comparing the results with those reported in honey from similar regions and with those reported by other authors. The pH values that were obtained in the analyzed samples ranged between 3.96 and 5.23; avocado honey showed the highest values and was similar to those reported in avocado honey from Spain [7,15]. The pH values in Eucalyptus honey were also within the range of values previously reported by this monofloral honey [9,16], while Rapeseed honey showed high acidity, being within the range of values previously reported [12].

European regulations set a maximum limit for electrical conductivity (EC) in blossom honey, below 0.8 mS/cm [13], however some monofloral honey such as chestnut, strawberry plant, heather, lime, and tea tree showed EC values above the limit that is established by current legislation [10,13]. According to the results here exposed, Avocado honey showed the highest values of EC, which was significant (*p* < 0.05) regarding the values found in Eucalyptus and Rapeseed honey and above the recommended limits for *Apis mellifera* honey (0.8 mS/cm). Our results are consistent with those previously reported for this monofloral honey from Spain with values higher than 0.8 Sm/cm [7,15], while Eucalyptus and Rapeseed honey showed EC values with the range suggested by international legislation [13]. A similar behaviour was observed in the ash content, where Avocado honey showed the highest ash content (*p* < 0.05) when compared to Eucalyptus and Rapeseed honey, even exceeding the recommended limits for *Apis mellifera* honey (0.6%) [13]. The ash values in Avocado honey reported here are in agreement with those previously reported in Avocado honey from Spain, the latter having values greater than 0.77% [15]. The EC in honey is related to concentration of mineral salts, organic acids, and proteins, showing great variability according to floral origin and representing an important parameter for differentiating honey according to floral origin [10]. A high correlation was found between EC and ash content, which could justify, in part, the high values found in the EC.

Hydroxymethylfurfural (HMF) and diastase index are used as indicators of honey freshness [13]. In fresh honey, HMF could be absent or low in quantity, while high HMF levels (˃80 mg/kg) may suggest inappropriate handling and storage conditions being influenced by factors, such as temperature and pH [17]. The HMF values in the three monofloral honey types analyzed were within the recommended ranges for *Apis mellifera* honey with declared origin from tropical climates, suggesting an appropriated processing, storage, and/or aging procedure by the beekeepers. According to international legislation, the diastase index must be above 8 Schade units [13]; lower values could be interpreted as aging or temperature abuse. According to the results obtained, all of the samples from the three monofloral honey types analyzed were within the recommended limits and were in line with the international regulation for these parameters [13].

### 2.2. Chemical Composition and Total Antioxidant Capacity (TAC)

The phytochemical and amino acid contents were also determined in the three monofloral honey types and results are shown in Table 1. Total phenolic content (TPC), total flavonoid content (TFC), and total carotenoid content (TCC) demonstrated significant differences (*p* < 0.05) between the three honey types: Avocado honey showed the highest values, while Rapeseed honey had the lowest values. These results are in agreement with those previously exposed by others authors who demonstrated that darker honeys present higher values of TPC, TFC, and TCC in comparison to light honeys [18]. A high correlation was found between colour vs. TPC, colour vs. TFC, and colour vs. TCC (Table 2).

Total free amino acid and proline contents were also analyzed and the results are shown in Table 1. The highest values of free amino acid and proline content were found in Avocado honey, followed by Eucalyptus and Rapeseed honey. The amino acid content in honey has been mainly associated with: (i) the introduction of certain enzymes by bees from their salivary glands and (ii) by the floral origin, nectar, and pollen collected from the plants visited by bees [19], which could explain, in part, the significant differences found between the different honeys. Despite these differences, the average values were within those previously reported by other authors, such as in Avocado honey [7] and Eucalyptus [9,20] from other geographical regions. No data was found about the chemical composition of Rapeseed honey, so, to the best of our knowledge, the results here exposed represent the first study into this monofloral honey.

**Figure 1:**
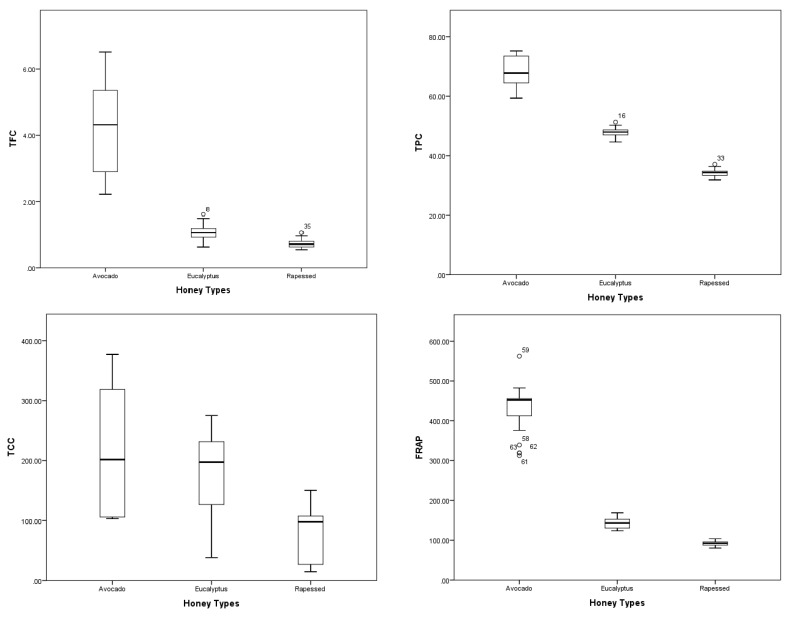

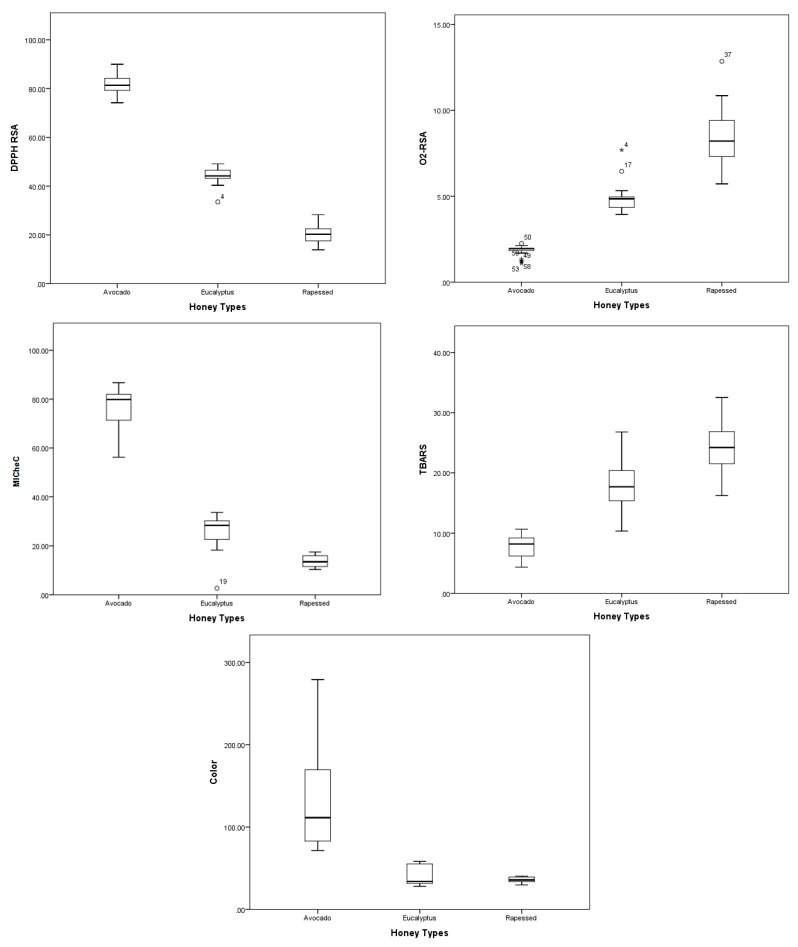
Box-plot diagrams of the levels of quantitative determinations in the three monofloral honeys. TPC, total phenolic content; TFC, total flavonoid content; TCC, total carotenoids content; FRAP, ferric reducing antioxidant power assay, DPPH RSA, DPPH radical scavenging activity; O_2_^•−^ RSA, superoxide anion radical scavenging activity, MICheC, metal ions chelating capacity.

The TAC in the three monofloral honey types was also determined and results are shown in Table 1. To determine the antioxidant capacity of honey samples, five different methods were used with the aim of studying this property through different mechanisms of action. An artificial honey solution was included in order to evaluate the contribution of the major sugars present in honey in the TAC and antimicrobial analysis [21]. In all of the TAC assays, the artificial honey solution showed significantly (*p* < 0.05) lower values as compared to the analyzed honey, which allows one to suppose that the contribution of the main sugar in the samples did not contribute significantly to their TAC. All of the honey samples were able to reduce Fe^3+^ to Fe^2+^, to scavenge the radical DPPH, and to chelate metal ions. Avocado honey showed the best results (*p* < 0.05) when compared to Eucalyptus and Rapeseed honey (Table 1). These results are in agreement with those previously reported by others authors who reported higher antioxidant capacity in dark honeys than in clear honeys [18,22]. The honey samples were also able to scavenge the O_2_^•−^ and to protect against lipid peroxidation in red blood cell membranes. Avocado honey was the most effective in scavenging the O_2_^•−^ and protecting against lipid peroxidation (*p* < 0.05), followed by Eucalyptus and Rapeseed honey. The O_2_^•^^−^ scavenging capacity of honey has been previously reported by others authors [23,24], as well as the protective effect against lipid peroxidation present in honey from different geographical and floral origins [23,25], which demonstrate the different mechanisms through which honey and its constituents, such as the polyphenols, exert their antioxidant capacity [26,27,28]. One can observe Figure 1 that TPC, TFC, TCC, TAC, metal ions chelating capacity, the superoxide radical scavenging activity (O_2_^•−^ RSA) and the protective effects against ghost membrane lipid peroxidation decrease from dark to white honeys, which is in line with the trend generally reported in the literature [18,22,23]. A significant correlation was found between colour, TPF and TFC vs. TAC assays (ferric reducing antioxidant power assay (FRAP), DPPH radical scavenging activity (DPPH RSA), O_2_^•−^ RSA, metal ions chelating capacity, and the prevention of lipid peroxidation) (Table 2), which is in agreement with what has been previously reported in honeys from diverse geographical and floral origins [18,29,30].

### 2.3. Effect of Honey to Inhibit Biofilm Formation and to Remove Preformed Biofilm in Staphylococcus aureus and Klebsiella pneumoniae Bacteria

The antimicrobial activity of honey has been attributed to two fundamental mechanisms. The first is the presence of a non-peroxide antibacterial activity, due to the high osmolarity and acidity of honey, as well as the presence of compounds with antimicrobial activity, such as methylglyoxal, bee defensing-1, and flavonoids. The second most important mechanism is related to the peroxide-associated activity due to the specific hydrogen peroxide content [2]. The antimicrobial activity of honey has been well documented and related to floral origin [31] and bee species [32,33], which has suggested a possible relationship between this biological property and the factors mentioned above.

In this sense, the capacity of the three monofloral honey types studied to reduce in vitro bacterial biofilms in two bacterial strains was assayed to determine the possible relationship between floral origin and antimicrobial capacity. Bacterial biofilm is usually established in open, chronic wounds prior to the patient’s presentation to a clinic for medical treatment, making its subsequent treatment difficult. Therefore, here, the capacity of the three monofloral honey to reduce preformed biofilm was studied, as well as its capacity to inhibit its formation using two bacterial strains: one gram-positive (*Staphylococcus aureus* CAMP) and one gram-negative (*Klebsiella pneumoniae* KPC 609803).

**Figure 2:**
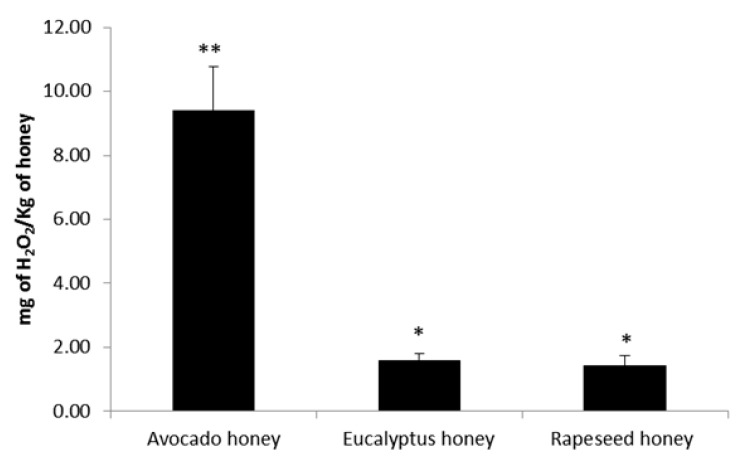
H_2_O_2_ values of the three monofloral honeys studied. Columns belonging to the same set of data with different symbols are significantly different. * *p* < 0.05 and ** *p* < 0.01.

Since the antimicrobial activity of honey has been closely related to peroxide-associated activity in honey [34], total hydrogen peroxide content in the honey samples was determined. H_2_O_2_ generation in honey is a result of glucose oxidation, which is catalyzed by glucose oxidase (GOX) that comes from the hypopharyngeal glands of honeybees [35], which is then added to the nectar by honeybees [34]. However, the accumulation of H_2_O_2_ in honey is dependent on several factors that have an impact on H_2_O_2_ production or neutralization, such as those that may affect GOX activity (temperature, floral origin, and health of bees) [36], and pollen-derived catalase that effectively hydrolyze H_2_O_2_ to oxygen and water, which are considered as a potent blocker of H_2_O_2_ accumulation [37]. The H_2_O_2_ values found in Eucalyptus and Rapeseed honey were within the ranges that were previously reported in monofloral honey form China [38], while the values found in Avocado honey were significantly higher (*p <* 0.01) than the Eucalyptus and Rapeseed honey (Figure 2). Therefore, taking into account the factors mentioned above, it is possible to explain, in some ways, the differences in values found in the studied honey types.

**Figure 3:**
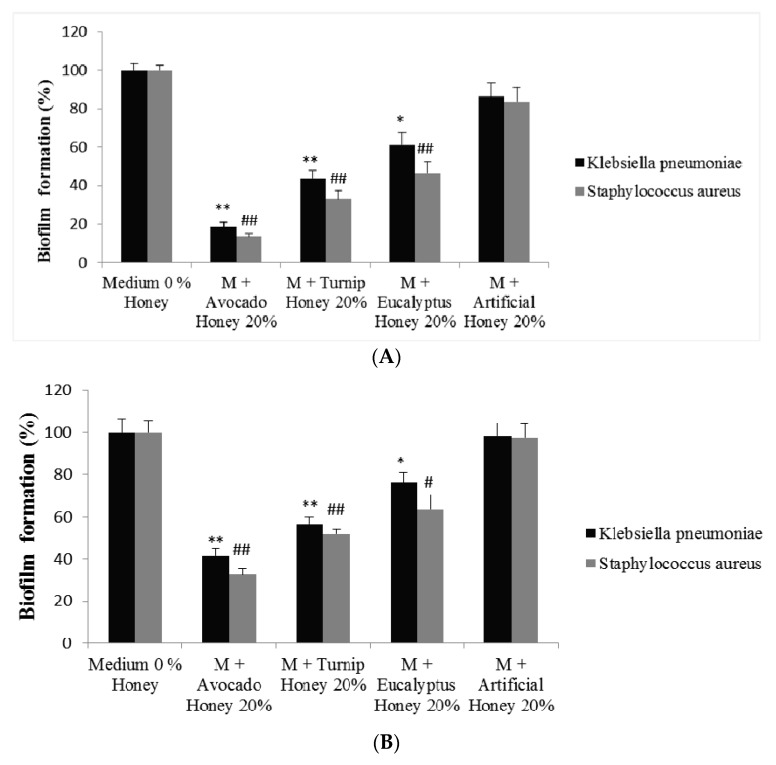
Effectiveness of the three monofloral honeys in inhibiting biofilm formation (**A**) and removing preformed biofilm (**B**) in *Staphylococcus aureus* and *Klebsiella pneumoniae* bacteria. Columns belonging to the same set of data with different symbols are significantly different when compared to control (Medium 0% Honey). * *p* < 0.05, ^#^ *p* < 0.05 and ** *p* < 0.05, ^##^ *p* < 0.01.

A similar behaviour was found in the capacity of the different honey types to inhibit biofilm formation and to remove preformed biofilm. Avocado honey (20% *w*/*v*) was more efficient in inhibiting biofilm formation (Figure 3A) and removing preformed biofilm (*p* > 0.01) (Figure 3B), followed by Rapeseed and Eucalyptus honey. The capacity of honey to affect bacterial biofilm has been previously reported [39,40] and has even been related to its floral origin [4]. Avocado and Rapeseed honey’s effectiveness against bacterial biofilm is inadequately reported, so, to the best our knowledge, the results here exposed represent the first report in this honey type. As previously mentioned, hydrogen peroxide is considered to be largely responsible for the antimicrobial properties of honey [34]; however, other compounds, such as polyphenols, have also been related to this biological property [41]. Avocado honey showed the highest values of TPC and hydrogen peroxide, therefore, these results could serve to justify, in part, the high antimicrobial capacity found in this monofloral honey in comparison to the others; however, other tests are necessary to confirm this statement.

## 3. Materials and Methods

### 3.1. Honey Samples

Three different types of monofloral honey were collected during 2015 and 2016 in the provinces of Pichincha, Cotopaxi, Tungurahua, and Chimborazo, all being located in the Andean region of Ecuador. The floral sources were Avocado (*Persea americana*), Eucalyptus (*Eucalyptus* sp.), and Rapeseed (*Brassica napus*). Samples of honey were collected directly from beekeepers, according to the data provided by the Ecuadorian Agency for Agricultural Quality Assurance (AGROCALIDAD, Ecuador). A total of 12 beekeepers were chosen, on the basis of number of apiaries kept (≥10) and the existence of production of any of the three types of monofloral honey under study. For each beekeeper, all apiaries were sampled by taking five samples at random from each apiary. The samples were kept at room temperature and the different analyses were performed within a time period not exceeding three months from the date of collection. The results were expressed as the mean values obtained from the analyses of all the samples from each beekeeper by honey type.

The floral origin of honey samples was confirmed through the melissopalynologycal methods suggested for this type of study [42,43]. A solution of artificial honey was included to eliminate any interference that might occur from these compounds in the tests performed [21].

### 3.2. Physicochemical Analysis

Physicochemical determinations were performed according to the standardized methods proposed by the International Honey Commission [44]. Moisture was determined by refractometry at 20 °C, and results were expressed as % of moisture. Colour was determined using a Pfund colour grader Koehler, and the results were expressed as mm Pfund. Electrical conductivity was measured at 20 °C and results were expressed as mS/cm. Hydroxymethyl furfural (HMF) and diastase activity were determined spectrophotometrically and results were expressed as mg/kg and Gothe units per gram of honey, respectively. pH and free acidity were measured in a honey solution in ultrapure water using a pH meter, and free acidity values were expressed as meq/kg.

### 3.3. Chemical Composition

Total phenolic content (TPC) was determined using the Folin–Ciocalteu method [45]. Gallic acid was used to produce the calibration curve (0.5–3 mM) and results were expressed as mg of gallic acid equivalents (GAE) per kg of honey (mgGAE/kg of honey). Total flavonoid content (TFC) was determined using the aluminium chloride colorimetric method [46]. (+) Catechin was used to produce the calibration curve and results were expressed as (+)-Catechin equivalents (CE) per kg of honey (mg of CE/kg of honey). Free amino acid content was determined using the Cd-ninhydrin method [47]. For free amino acid, L-leucine was used for the calibration curve and results were expressed as mg of L-leucine equivalents (LE) per 100 g of honey (mg LE/100 g of honey), while for proline content, proline was used for the calibration curve and the results were expressed as mg of proline content (Prol) per 100 g of honey (mg Prol/100 g of honey). Total carotenoid content (TCC) was determined spectrophotometrically [29]. *β*-carotene was used for the calibration curve and results were expressed as mg of *β*-carotene equivalents (*β*carotE) per kg of honey (mg *β*carotE/kg of honey).

### 3.4. Total Antioxidant Capacity (TAC) Assays

#### FRAP, DPPH, Metal Ions Chelating Capacity, and the Superoxide Radical (O_2_^•−^) Scavenging Activity

TAC was determined spectrophotometrically using the FRAP [48] and DPPH assays [49]. Trolox was used for the calibration curves in both assays and the results were expressed as μmoles of Trolox equivalents per 100 g of honey (μmol TE/100 g of honey).

Chelating metal ions capacity was determined as previously reported [50]. In short, 0.05 mL of FeCl_2_·4H_2_O (2 mM) was added to the honey solutions, and then the reaction was initiated by the subsequent addiction of 0.2 mL of ferrozine (5 mM). The reaction mixture was incubated for 10 min at room temperature and the absorbance was determined spectrophotometrically at 562 nm. Chelating ability results were expressed as percentages and were calculated using the following Equation:

ChA = [(A_0_ − A_1_)/A_0_] × 100 (1)

in which A_0_ was the absorbance of the control and A_1_ the absorbance of each honey sample.

The O_2_^•−^ scavenging activity of honey was investigated using a modified nitrite method that was based on the ability of the O_2_^•−^ generated by hypoxanhine/xanthine oxidase (HX/XO) to oxidize hydroxylamine to nitrite ion at pH 8.2 [51]. The nitrite ion generated was measured spectrophotometrically at 550 nm, and the results were expressed as the percentages of inhibition (PI) of nitrite ion generation in the presence of honey, as calculated by the change in the slope of increasing spectrophotometric absorption (A_m_) of the samples when compared to the blank (A_b_, with distilled H_2_O), using the Equation:

PI = [(A_b_ − A_m_)/A_b_] × 100 (2)

The O_2_^•−^ scavenging activity was expressed as the amount of honey able to inhibit the generation of the nitrite ion at 50% (IC_50_) (mg/mL).

### 3.5. Protective Effects against Ghost Membrane Lipid Peroxidation

#### 3.5.1. Blood Collection and Red Blood Cells (RBC) Ghost Membrane Preparation

RBC were obtained from healthy non-smoking adult volunteers after informed consent. Erythrocytes were isolated from heparinized blood by centrifugations at 1000× *g* for 10 min at 10 °C. Plasma and buffy coat were removed and RBC were washed once with cool 0.9% NaCl solution and three times with PBS (150 mM NaCl, 1.9 mM Na_2_HPO_4_ and 8.1 mM NaH_2_PO_4_, pH 7.4) at 4 °C.

Ghost membrane preparation was performed as previously reported [52]. To produce complete hemolysis, RBC were washed with hypotonic solutions at different concentrations as follows: firstly, RBC were re-suspended in 20 times their volume (1:20), with 5 mM of hypotonic phosphate buffer (2.2 mM EDTA, 1:20) at pH 7.4 and kept at 4 °C for 30 min. After incubation, the solution was centrifuged at 3800× *g* for 20 min at 8 °C, the supernatant was discarded and the pellet re-suspended in 2.5 mM of hypotonic phosphate buffer (1:20) pH 8. In the final stage, 1.25 mM of phosphate buffer pH 8 was added and centrifuged at 13,500 rpm for 20 min at 8 °C. At the end of the procedure, RBC ghost membranes in the pellet appeared completely clear. Ghost membranes were then re-suspended in PBS buffer plus 2.2 mM of EDTA (1:5) and stored at −80 °C until analysis. Membrane protein was determined by the Lowry method [53].

#### 3.5.2. Determination of Lipid Peroxidation in Ghost Membrane

Prior to the test, RBC ghost membranes were thawed and the storage buffer was removed by centrifugation (1800× *g* for 20 min at 8 °C). Pellets were washed twice in 0.9% NaCl and the packed ghost membranes were then re-suspended in 0.9% NaCl for the analysis.

The protective effect of honey samples against RBC ghost membrane lipid peroxidation was determined as follows:

The reaction mixture (50 μL of honey solution (40–5 mg/mL), 50 μL of AAPH (50 mM) and 100 μL of RBC ghost membranes) was incubated at 37 °C for 60 min and then the reaction was stopped. TBARS formation was determined as a measure of lipid peroxidation after RBC ghost membranes were exposed to the oxidant using a modified thiobarbituric acid (TBA) assay [54]. Briefly, 1 mL of TBA–TCA–HCl (0.375% *w*/*v* TBA, 15% *w*/*v* TCA, 0.2 M HCl) solution was added to 0.5 mL of sample containing 0.3 mM BHT to prevent the possible peroxidation of ghost membrane lipids during the TBA assay. The samples were heated for 20 min at 95 °C and then cooled at room temperature and centrifuged (14,000 rpm) for 10 min, and the absorbance of the supernatant was measured spectrophotometrically at 535 nm (SynergyTM Multi-Detection Microplate Reader; Bio-Tek^®^, Instruments, Inc., Winooski, VT, USA). One control (i) was included to eliminate the basal lipid peroxidation that was present in the tissue (C_0_) and a second control (ii) to determine the maximum values of lipid peroxidation in each assay (C_60_). The antioxidant activity of the samples was expressed as the percentage inhibition (PI) of the formation of TBARS that was produced by honey from their absorbance value (A_m_) when compared to controls, and was calculated using the equation:

PI = [1 − (A_m_ − C_0_)/(C_60_ − C_0_)] × 100 (3)

The results are expressed as the concentration of honey that causes 50% inhibition of maximum formation of TBARS (IC_50_) in the ghost membrane.

### 3.6. Antimicrobial Activity

#### 3.6.1. Hydrogen Peroxide Content in Honey Samples

Hydrogen peroxide content was determined using the modified FOX-1 method for the micro-determination of hydrogen peroxide in honey samples, as recently reported [38]. The FOX-1 working solution was a freshly prepared mixture of 25 mM of ammonium ferrous sulphate in 0.25 M of sulphuric acid with a solution consisting of 62.5 L M xylenol orange and 150 mM sorbitol at a ratio of 1:100. Then, 80 μL of a properly diluted honey was added to 160 μL of the FOX-1 working solution. This mixture was incubated in the dark for 30 min, then the absorbance was read at 580 nm using an automated 96-well microplate reader (SynergyTM Multi-Detection Microplate Reader; Bio-Tek^®^, Instruments, Inc., Winooski, VT, USA). For each analysis, the background response of the FOX-1 reagent was subtracted out by using distilled water as a control. H_2_O_2_ standard stock solution was prepared by diluting a H_2_O_2_ solution (30%) to give a final concentration of 3.4 mg/mL. To prepare the calibration curve, varying amounts of a 5000-fold diluted H_2_O_2_ stock solution were transferred into a 96-well plate and completed to a final volume of 80 μL with diluted water to obtain H_2_O_2_ dilution ranges from 0.0136 μg/mL to 0.68 μg/mL. Aliquots (160 μL) of freshly prepared FOX-1 reagent were then added to each well, incubated for 30 min, and read at 580 nm using an automated 96-well microplate reader. Hydrogen peroxide content in honey samples was expressed as μg/kg of honey.

#### 3.6.2. Determination of the Ability of Honey to Inhibit Biofilm Formation and Remove Preformed Biofilm

The capacity of honey solutions to inhibit biofilm formation was determinated using the biofilm formation assay in a microtiter plate outlined by published studies [39]. Two bacterial strains: one gram-positive (*Staphylococcus aureus* CAMP) and one gram-negative (*Klebsiella pneumoniae* KPC 609803) were used in the assay. Bacterial strains were cultured in TBS (Tryptic Soy Broth) and shaken (90 rpm) at 37 °C for 18 h The culture suspension was diluted at 0.5-fold on the McFarland scale (1.5 × 10^8^ UFC) and 100 μL of this dilution was transferred into a 96-well plate. Then, 100 μL of TBS containing the appropriated test solution (Avocado, Rapeseed, Eucalyptus and Artificial honey at 20 and 10%) was added and the plate was incubated for 24 h at 37 °C. Media and media with the solution test without bacteria inoculation were used as negative controls. Following this step, planktonic cell growth was eliminated and the plates were treated appropriately for the spectrophotometric quantification of the adhered biofilm. Biofilm formation was expressed as a percentage relative to that produced by the untreated control, which was set as 100%.

To determine the ability of honey to remove preformed biofilm, biofilms were first formed in the 96-well microtiter plate for 24 h at 37 °C, as described above. These biofilms were then washed three times with PBS and incubated with the appropriated test solution (Avocado, Rapeseed, Eucalyptus, and Artificial honey at 20% and 10%) for a further 24 h at 37 °C. Planktonic cell was eliminated and biofilm mass was quantified and expressed, as described above.

### 3.7. Statistical Analyses

The samples were analyzed in triplicate and the results were expressed as mean ± standard deviation (SD). Statistical analyses were performed using IBM SPSS Statistics for Windows version 20.0. One-way ANOVA was used to determine the significant differences between samples using a Bonferroni correction for multiple sample comparison. The correlations between the variables were calculated using Pearson’s coefficient. In all of the cases, a *p*-value < 0.05 was considered as statistically significant.

## 4. Conclusions

In conclusion, this paper contains key information about the relationship between the floral origin and chemical and biological properties of monofloral honey from the Andean regions of Ecuador. To the best of our knowledge, there are few reports about the biological properties and the chemical composition of honey produced at high altitudes (above 2500 m above sea level), as well as about the physicochemical and biological properties of Rapeseed honey. The chemical and biological properties of the three honey types were well defined in terms of floral origin and their relationship with total bioactive compounds content (TPC, TFC, TCC), as well as their antioxidant capacity, demonstrating important abilities in scavenging free radicals and protecting against oxidative damage to lipids. On the other hand, the antimicrobial capacity of each type of honey and its possible relationship with floral origin were also evidenced. The findings presented in this research could provide new knowledge to complement that which already exists on the antimicrobial properties of honey, especially from the point of the relationship between its floral origin and antimicrobial capacity. Therefore, honey could be proposed as an alternative treatment for opportunistic infections that are caused by different germs in soft tissue and surgical wound infections. All of this, together with its ability to promote wound healing and its anti-inflammatory and antioxidant capacity could be an efficient clinical alternative for the treatment of wounds. On the other hand, this initial study about the chemical and biological properties of monofloral honey types from the Andean regions of Ecuador indicates that they have comparable or superior potential benefits to the monofloral honey from other floral and geographical origins, justifying their use as a natural source of bioactive compounds with important biological properties for humans and animals.

## Figures and Tables

**Table 1:** Physicochemical parameters, bioactive compound and total antioxidant capacity in monofloral honeys from Ecuador.

Parameters	Monofloral Honey Types/Values		
	Avocado Honey	Eucalyptus Honey	Rapeseed Honey
**Physicochemical parameters**			
Colour (mm Pfund)	137.29 ± 66.97 ^a^	39.87 ± 11.46 ^b^	37.09 ±3.32 ^b^
Moisture (%)	16.42 ± 2.53 ^a^	18.62 ± 1.84 ^b^	14.63 ± 2.74 ^c^
pH	5.23 ± 0.96 ^a^	4.01 ± 0.18 ^b^	3.96 ± 0.24 ^b^
Hydroxymethylfurfural (mg/kg of honey)	27.16 ± 30.43 ^a^	3.78 ± 3.48 ^b^	70.81 ± 7.86 ^c^
Diastase index (ºGothe)	47.65 ± 34.95 ^a^	32.92 ± 11.35 ^b^	13.71 ± 2.99 ^c^
Electrical conductivity (mS/cm)	1.31 ± 0.47 ^a^	0.39 ± 0.03 ^b^	0.20 ± 0.01 ^c^
Ash content (%)	1.05 ± 0.13 ^a^	0.25 ± 0.02 ^b^	0.11 ± 0.01 ^c^
**Bioactive compounds**			
Total phenolic content (mg GAE/100 g of honey)	68.23 ± 5.79 ^a^	47.71 ± 1.71 ^b^	34.33 ± 0.895 ^c^
Total flavonoid content (mg CEq/100 g of honey)	4.25 ± 1.22 ^a^	1.09 ± 0.24 ^b^	0.72 ± 0.20 ^c^
Total carotenoids content (mg *β*carotE/kg of honey)	2.24 ± 0.67 ^a^	1.60 ± 0.33 ^b^	0.76 ± 0.14 ^c^
Total free amino acids content (mg LE/100 g of honey)	83.46 ± 43.41 ^a^	14.07 ± 3.20 ^b^	10.53 ± 3.66 ^c^
Total proline content (mg Prol/100 g of honey)	1039.94 ± 53.31 ^a^	140.82 ± 40.71 ^b^	88.19 ± 18.53 ^c^
**Total antioxidant capacity (TAC)**			
FRAP (μmol TE/100 g of honey)	425.35 ± 49.24 ^a^	142.97 ± 13.84 ^b^	92.05 ± 4.30 ^c^
DPPH (μmol TE/100 g of honey)	84.05 ± 5.16 ^a^	44.30 ± 5.33 ^b^	18.22 ± 3.19 ^c^
O_2_^•^^−^ Scavenging activity IC_50_ (mg/mL)	1.82 ± 0.32 ^a^	4.90 ± 0.84 ^b^	8.42 ± 1.72
Chelating metal ions capacity (%)	76.90 ± 7.08 ^a^	26. 17 ± 6.92 ^b^	13.69 ± 2.35 ^c^
TBARS assay IC_50_ (mg/mL)	7.59 ± 1.85 ^a^	17.78 ± 3.97 ^b^	24.40 ± 4.98 ^c^

Sample was analyzed in triplicate and data are presented as means ± standard deviation. ^a,b,c^ represent significant difference for *p* < 0.05. GAE, Gallic Acid Equivalents; CEq, Catechin Equivalents; *β*carotE, *β*-carotene Equivalents; LE, L-leucine Equivalents; Prol, Proline; FRAP, Ferric Reducing Antioxidant Power; O_2_^•−^, RSA, Superoxide Radical Scavenging Activity; TBARS, Thiobarbituric Acid Reactive Substances; TE, Trolox equivalents; DPPH, DPPH radical scavenging activity.

**Table 2:** Correlation matrix for quantitative determinations in the three monofloral honeys.

Variables	Colour	TPC	TFC	TCC	FRAP	DPPH RSA	O_2_^•−^ RSA	MICheC	TBARS
**Colour**	-	0.770 **	0.860 **	0.378 *	0.787 **	0.729 **	−0.613 **	0.716 **	−0.699 **
**TPC**	-	-	0.905 **	0.688 **	0.937 **	0.958 **	−0.890 **	0.921 **	−0.880 **
**TFC**	-	-	-	0.625 **	0.899 **	0.820 **	−0.734 **	0.845 **	−0.782 **
**TCC**	-	-	-	-	0.572 **	0.609 **	−0.596 **	0.481 **	−0.481 **
**FRAP**	-	-	-	-	-	0.932 **	−0.827 **	0.943 **	−0.847 **
**DPPH RSA**	-	-	-	-	-	-	−0.899 **	0.944 **	−0.884 **
**O_2_^•−^ RSA**	-	-	-	-	-	-	-	−0.837 **	0.840 **
**MICheA**	-	-	-	-	-	-	-	-	−0.869 **
**TBARS**	-	-	-	-	-	-	-	-	-

Except in the diagonal, the values at the level of significance are reported. TPC, total phenolic content; TFC, total flavonoid content; TCC, total carotenoids content; FRAP, ferric reducing antioxidant power assay, DPPH RSA, DPPH radical scavenging activity; O_2_^•−^ RSA, superoxide anion radical scavenging activity, MICheC, metal ions chelating capacity. * Significant at *p* < 0.05; ** Significant at *p* > 0.01.

## Abbreviations

HMF	Hydroxymethylfurfural
TPC	Total phenolic content
TFC	Total flavonoid content
TCC	Total carotenoid content
TAC	Total antioxidant capacity
FRAP	Ferric reducing antioxidant power
O_2_^•−^ RSA	Superoxide radical scavenging activity
TBARS	Thiobarbituric acid reactive substances
GAE	Gallic acid equivalents
CEq	Catechin equivalents
LE	Leucine equivalents
Prol	Proline
RBCs	Red blood cells
